# 1,3-Bis[4-(4-pyrid­yl)pyrimidin-2-ylsulfan­yl]propane

**DOI:** 10.1107/S1600536808010854

**Published:** 2008-04-23

**Authors:** Yan-Yan Sun, Hua-Ze Dong, Lin Cheng

**Affiliations:** a Department of Chemistry and Chemical Engineering, Southeast University, Nanjing, People’s Republic of China; b Department of Chemistry and Chemical Engineering, State Key Laboratory of Coordination Chemistry, Nanjing University, Nanjing, People’s Republic of China

## Abstract

In the title compound, C_21_H_18_N_6_S_2_, the dihedral angles between the aromatic rings in the two 4-(4-pyrid­yl)pyrimidine residues are 23.45 (13) and 2.67 (14)°. Whereas one of the C—S—C—C torsion angles corresponds to a staggered conformation, the other is gauche.

## Related literature

For related structures, see: Awaleh *et al.* (2005 ▶); Xie *et al.* (2005 ▶).
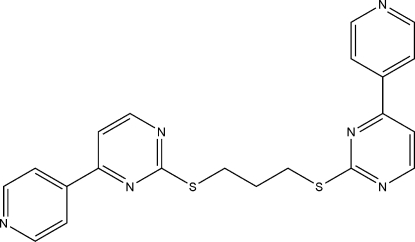


## Experimental

### 

#### Crystal data

C_21_H_18_N_6_S_2_
*M* *_r_* = 418.55
Triclinic, 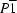

*a* = 9.986 (3) Å
*b* = 10.057 (3) Å
*c* = 10.645 (3) Å
α = 98.972 (5)°
β = 90.688 (5)°
γ = 112.632 (5)°
*V* = 971.6 (5) Å^3^
*Z* = 2
Mo *K*α radiation
μ = 0.29 mm^−1^
*T* = 291 (2) K
0.30 × 0.20 × 0.20 mm

#### Data collection

Bruker SMART CCD area-detector diffractometer
Absorption correction: multi-scan (*SADABS*; Bruker, 2000 ▶) *T*_min_ = 0.915, *T*_max_ = 0.943
5163 measured reflections
3720 independent reflections
2986 reflections with *I* > 2σ(*I*)
*R*_int_ = 0.028

#### Refinement

*R*[*F* ^2^ > 2σ(*F* ^2^)] = 0.059
*wR*(*F* ^2^) = 0.156
*S* = 1.02
3720 reflections
262 parameters
H-atom parameters constrained
Δρ_max_ = 0.52 e Å^−3^
Δρ_min_ = −0.46 e Å^−3^

### 

Data collection: *SMART* (Bruker, 2000 ▶); cell refinement: *SMART*; data reduction: *SAINT* (Bruker, 2000 ▶); program(s) used to solve structure: *SHELXTL* (Sheldrick, 2008 ▶); program(s) used to refine structure: *SHELXTL*; molecular graphics: *SHELXTL*; software used to prepare material for publication: *SHELXTL*.

## Supplementary Material

Crystal structure: contains datablocks I, global. DOI: 10.1107/S1600536808010854/bt2695sup1.cif

Structure factors: contains datablocks I. DOI: 10.1107/S1600536808010854/bt2695Isup2.hkl

Additional supplementary materials:  crystallographic information; 3D view; checkCIF report

## Figures and Tables

**Fig. 1.**
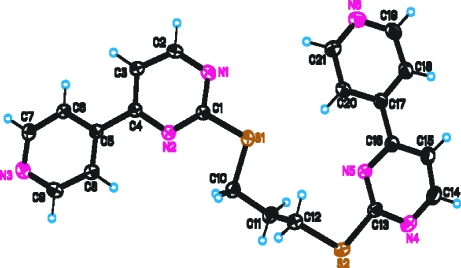
The molecular structure of the title compound.

## supplementary crystallographic information

### Comment

In the title compound,   the dihedral angles between the aromatic rings in the two(4-pyridyl)-pyrimidine residues are 23.45 (13)° and 2.67 (14)°. Whereas one of the C-S-C-C torsion angles adopts a staggered conformation [-176.74 (17)°], the other one is gauche [87.6 (2)°].

### Experimental

NaOH (0.80 g, 20 mmol) in ethanol (10 mL) was added with stirring to a solution of 4-(pyridin-4-yl)pyrimidine-2-thiol (3.78 g, 20 mmol) in acetone (30 mL) at ambient temperature for 20 min. A solution of 1,3-dibromopropane (2.00 g, 10.0 mmol) was added slowly over 1 h, and the resulting mixture was further refluxed for 6 h. The solids isolated from the reaction mixture were washed with water and acetone. The yellow products were obtained by vacuum dryness in 91% yield (3.80 g). The filtrate was allowed to evaporate slowly at room temperature. After several days, yellow block shaped crystals suitable for X-ray diffraction analyses were obtained.

### Refinement

H atoms were positioned geometrically and refined using a riding model, with C—H = 0.93 and 0.97 Å for C_aromatic_ and C_methylene_, respectively, and with *U*_iso_(H) = 1.2*U*_eq_(C).

### Crystal data

**Table:**

C_21_H_18_N_6_S_2_	*Z* = 2
*M**_r_* = 418.55	*F*_000_ = 436.0
Triclinic, *P*1	*D*_x_ = 1.431 Mg m^−^^3^
Hall symbol: -P 1	Mo *K*α radiation λ = 0.71073 Å
*a* = 9.986 (3) Å	Cell parameters from 765 reflections
*b* = 10.057 (3) Å	θ = 2.5–28.0º
*c* = 10.645 (3) Å	µ = 0.30 mm^−^^1^
α = 98.972 (5)º	*T* = 291 (2) K
β = 90.688 (5)º	Block, yellow
γ = 112.632 (5)º	0.30 × 0.20 × 0.20 mm
*V* = 971.6 (5) Å^3^	

### Data collection

**Table:**

Bruker CCD area-detector diffractometer	3720 independent reflections
Radiation source: fine-focus sealed tube	2986 reflections with *I* > 2σ(*I*)
Monochromator: graphite	*R*_int_ = 0.028
*T* = 291(2) K	θ_max_ = 26.0º
φ and ω scans	θ_min_ = 1.9º
Absorption correction: multi-scan(SADABS; Bruker, 2000)	*h* = −12→11
*T*_min_ = 0.915, *T*_max_ = 0.943	*k* = −12→10
5163 measured reflections	*l* = −12→13

### Refinement

**Table:**

Refinement on *F*^2^	Secondary atom site location: difference Fourier map
Least-squares matrix: full	Hydrogen site location: inferred from neighbouring sites
*R*[*F*^2^ > 2σ(*F*^2^)] = 0.059	H-atom parameters constrained
*wR*(*F*^2^) = 0.156	*w* = 1/[σ^2^(*F*_o_^2^) + (0.1031*P*)^2^] where *P* = (*F*_o_^2^ + 2*F*_c_^2^)/3
*S* = 1.02	(Δ/σ)_max_ < 0.001
3720 reflections	Δρ_max_ = 0.52 e Å^−^^3^
262 parameters	Δρ_min_ = −0.46 e Å^−^^3^
Primary atom site location: structure-invariant direct methods	Extinction correction: none

### Special details

**Table:**

Geometry. All e.s.d.'s (except the e.s.d. in the dihedral angle between two l.s. planes) are estimated using the full covariance matrix. The cell e.s.d.'s are taken into account individually in the estimation of e.s.d.'s in distances, angles and torsion angles; correlations between e.s.d.'s in cell parameters are only used when they are defined by crystal symmetry. An approximate (isotropic) treatment of cell e.s.d.'s is used for estimating e.s.d.'s involving l.s. planes.
Refinement. Refinement of *F*^2^ against ALL reflections. The weighted *R*-factor *wR* and goodness of fit *S* are based on *F*^2^, conventional *R*-factors *R* are based on *F*, with *F* set to zero for negative *F*^2^. The threshold expression of *F*^2^ > σ(*F*^2^) is used only for calculating *R*-factors(gt) *etc*. and is not relevant to the choice of reflections for refinement. *R*-factors based on *F*^2^ are statistically about twice as large as those based on *F*, and *R*- factors based on ALL data will be even larger.

### Fractional atomic coordinates and isotropic or equivalent isotropic displacement parameters (Å^2^)

**Table:**

	*x*	*y*	*z*	*U*_iso_*/*U*_eq_	
S1	0.66814 (7)	0.18543 (8)	0.02844 (6)	0.0501 (2)	
S2	0.31122 (8)	0.33035 (8)	0.24761 (7)	0.0551 (2)	
N1	0.9037 (2)	0.1412 (2)	−0.0039 (2)	0.0484 (5)	
N2	0.88209 (19)	0.3272 (2)	−0.10815 (17)	0.0362 (4)	
N3	1.1505 (2)	0.6455 (2)	−0.4191 (2)	0.0517 (6)	
N4	0.1388 (2)	0.1026 (2)	0.3337 (2)	0.0516 (5)	
N5	0.3748 (2)	0.1065 (2)	0.29548 (17)	0.0394 (5)	
N6	0.6716 (3)	−0.1928 (3)	0.3320 (2)	0.0558 (6)	
C1	0.8365 (2)	0.2254 (3)	−0.0352 (2)	0.0374 (5)	
C2	1.0326 (3)	0.1682 (3)	−0.0486 (2)	0.0488 (6)	
H2	1.0859	0.1158	−0.0270	0.059*	
C3	1.0916 (3)	0.2709 (3)	−0.1260 (2)	0.0449 (6)	
H3	1.1826	0.2879	−0.1565	0.054*	
C4	1.0107 (2)	0.3474 (2)	−0.1565 (2)	0.0346 (5)	
C5	1.0584 (2)	0.4510 (2)	−0.2471 (2)	0.0355 (5)	
C6	1.1556 (3)	0.4399 (3)	−0.3360 (2)	0.0438 (6)	
H6	1.1925	0.3676	−0.3397	0.053*	
C7	1.1961 (3)	0.5387 (3)	−0.4185 (3)	0.0520 (7)	
H7	1.2605	0.5294	−0.4784	0.062*	
C8	1.0070 (3)	0.5595 (3)	−0.2489 (2)	0.0423 (6)	
H8	0.9392	0.5687	−0.1928	0.051*	
C9	1.0560 (3)	0.6538 (3)	−0.3333 (3)	0.0504 (6)	
H9	1.0216	0.7279	−0.3311	0.061*	
C10	0.6054 (3)	0.3160 (3)	−0.0212 (2)	0.0454 (6)	
H10A	0.6765	0.4146	0.0080	0.055*	
H10B	0.5920	0.2998	−0.1135	0.055*	
C11	0.4614 (3)	0.2964 (3)	0.0364 (2)	0.0475 (6)	
H11A	0.4109	0.3412	−0.0101	0.057*	
H11B	0.4011	0.1928	0.0263	0.057*	
C12	0.4802 (3)	0.3627 (3)	0.1758 (2)	0.0446 (6)	
H12A	0.5352	0.4674	0.1852	0.054*	
H12B	0.5367	0.3228	0.2213	0.054*	
C13	0.2733 (3)	0.1608 (3)	0.2964 (2)	0.0416 (5)	
C14	0.1092 (3)	−0.0215 (3)	0.3770 (2)	0.0519 (7)	
H14	0.0168	−0.0673	0.4037	0.062*	
C15	0.2062 (3)	−0.0862 (3)	0.3847 (2)	0.0464 (6)	
H15	0.1824	−0.1720	0.4179	0.056*	
C16	0.3416 (3)	−0.0181 (2)	0.3409 (2)	0.0373 (5)	
C17	0.4557 (3)	−0.0781 (2)	0.3402 (2)	0.0373 (5)	
C18	0.4364 (3)	−0.2049 (3)	0.3858 (2)	0.0491 (6)	
H18	0.3500	−0.2550	0.4204	0.059*	
C19	0.5447 (3)	−0.2568 (3)	0.3800 (2)	0.0546 (7)	
H19	0.5286	−0.3423	0.4117	0.066*	
C20	0.5885 (3)	−0.0082 (3)	0.2938 (2)	0.0443 (6)	
H20	0.6092	0.0798	0.2650	0.053*	
C21	0.6903 (3)	−0.0697 (3)	0.2905 (3)	0.0536 (7)	
H21	0.7781	−0.0217	0.2569	0.064*	

### Atomic displacement parameters (Å^2^)

**Table:**

	*U*^11^	*U*^22^	*U*^33^	*U*^12^	*U*^13^	*U*^23^
S1	0.0461 (4)	0.0571 (4)	0.0600 (4)	0.0260 (3)	0.0245 (3)	0.0303 (3)
S2	0.0617 (5)	0.0605 (4)	0.0689 (5)	0.0449 (4)	0.0295 (4)	0.0283 (4)
N1	0.0516 (13)	0.0521 (12)	0.0558 (13)	0.0287 (10)	0.0163 (10)	0.0272 (10)
N2	0.0346 (10)	0.0392 (10)	0.0392 (10)	0.0173 (8)	0.0077 (8)	0.0118 (8)
N3	0.0481 (13)	0.0537 (13)	0.0615 (14)	0.0215 (10)	0.0138 (10)	0.0283 (11)
N4	0.0447 (12)	0.0597 (14)	0.0598 (13)	0.0287 (11)	0.0183 (10)	0.0142 (11)
N5	0.0438 (11)	0.0451 (11)	0.0400 (10)	0.0264 (9)	0.0124 (8)	0.0134 (8)
N6	0.0701 (16)	0.0584 (13)	0.0590 (14)	0.0434 (12)	0.0147 (12)	0.0192 (11)
C1	0.0389 (12)	0.0410 (12)	0.0381 (12)	0.0193 (10)	0.0083 (10)	0.0132 (10)
C2	0.0470 (14)	0.0536 (15)	0.0620 (16)	0.0306 (12)	0.0109 (12)	0.0270 (12)
C3	0.0391 (13)	0.0505 (14)	0.0575 (15)	0.0257 (11)	0.0139 (11)	0.0226 (12)
C4	0.0326 (11)	0.0355 (11)	0.0385 (12)	0.0148 (9)	0.0052 (9)	0.0103 (9)
C5	0.0296 (11)	0.0382 (12)	0.0414 (12)	0.0145 (9)	0.0036 (9)	0.0113 (9)
C6	0.0381 (13)	0.0482 (14)	0.0553 (14)	0.0232 (11)	0.0144 (11)	0.0206 (11)
C7	0.0458 (15)	0.0595 (16)	0.0604 (16)	0.0242 (13)	0.0210 (12)	0.0277 (13)
C8	0.0409 (13)	0.0445 (13)	0.0485 (13)	0.0219 (11)	0.0099 (10)	0.0139 (11)
C9	0.0516 (15)	0.0439 (14)	0.0654 (16)	0.0244 (12)	0.0104 (13)	0.0218 (12)
C10	0.0450 (14)	0.0548 (15)	0.0446 (13)	0.0251 (12)	0.0125 (11)	0.0164 (11)
C11	0.0392 (13)	0.0628 (16)	0.0494 (14)	0.0265 (12)	0.0092 (11)	0.0172 (12)
C12	0.0447 (14)	0.0477 (13)	0.0508 (14)	0.0251 (11)	0.0122 (11)	0.0160 (11)
C13	0.0477 (14)	0.0485 (13)	0.0392 (12)	0.0294 (11)	0.0123 (10)	0.0097 (10)
C14	0.0455 (15)	0.0552 (15)	0.0556 (15)	0.0197 (12)	0.0189 (12)	0.0098 (12)
C15	0.0501 (15)	0.0438 (13)	0.0455 (14)	0.0172 (12)	0.0153 (11)	0.0109 (11)
C16	0.0441 (13)	0.0399 (12)	0.0309 (11)	0.0200 (10)	0.0073 (9)	0.0057 (9)
C17	0.0488 (14)	0.0389 (12)	0.0299 (11)	0.0226 (10)	0.0091 (9)	0.0077 (9)
C18	0.0627 (16)	0.0483 (14)	0.0488 (14)	0.0303 (13)	0.0199 (12)	0.0201 (11)
C19	0.080 (2)	0.0489 (15)	0.0537 (15)	0.0400 (14)	0.0211 (14)	0.0233 (12)
C20	0.0506 (15)	0.0425 (13)	0.0508 (14)	0.0256 (11)	0.0134 (11)	0.0193 (11)
C21	0.0560 (16)	0.0590 (16)	0.0627 (16)	0.0353 (14)	0.0207 (13)	0.0242 (13)

### Geometric parameters (Å, °)

**Table:**

S1—C1	1.743 (2)	C7—H7	0.9300
S1—C10	1.799 (3)	C8—C9	1.365 (3)
S2—C13	1.762 (3)	C8—H8	0.9300
S2—C12	1.796 (2)	C9—H9	0.9300
N1—C2	1.320 (3)	C10—C11	1.525 (3)
N1—C1	1.342 (3)	C10—H10A	0.9700
N2—C1	1.325 (3)	C10—H10B	0.9700
N2—C4	1.343 (3)	C11—C12	1.507 (3)
N3—C7	1.320 (3)	C11—H11A	0.9700
N3—C9	1.338 (3)	C11—H11B	0.9700
N4—C14	1.325 (3)	C12—H12A	0.9700
N4—C13	1.336 (3)	C12—H12B	0.9700
N5—C13	1.323 (3)	C14—C15	1.367 (4)
N5—C16	1.337 (3)	C14—H14	0.9300
N6—C21	1.326 (3)	C15—C16	1.385 (3)
N6—C19	1.330 (4)	C15—H15	0.9300
C2—C3	1.379 (3)	C16—C17	1.480 (3)
C2—H2	0.9300	C17—C18	1.381 (3)
C3—C4	1.379 (3)	C17—C20	1.380 (3)
C3—H3	0.9300	C18—C19	1.368 (4)
C4—C5	1.478 (3)	C18—H18	0.9300
C5—C8	1.376 (3)	C19—H19	0.9300
C5—C6	1.386 (3)	C20—C21	1.377 (4)
C6—C7	1.376 (3)	C20—H20	0.9300
C6—H6	0.9300	C21—H21	0.9300
			
C1—S1—C10	103.68 (11)	H10A—C10—H10B	108.4
C13—S2—C12	102.41 (11)	C12—C11—C10	113.0 (2)
C2—N1—C1	114.9 (2)	C12—C11—H11A	109.0
C1—N2—C4	115.73 (19)	C10—C11—H11A	109.0
C7—N3—C9	115.9 (2)	C12—C11—H11B	109.0
C14—N4—C13	114.3 (2)	C10—C11—H11B	109.0
C13—N5—C16	116.7 (2)	H11A—C11—H11B	107.8
C21—N6—C19	115.5 (2)	C11—C12—S2	113.52 (17)
N2—C1—N1	127.7 (2)	C11—C12—H12A	108.9
N2—C1—S1	119.77 (17)	S2—C12—H12A	108.9
N1—C1—S1	112.50 (17)	C11—C12—H12B	108.9
N1—C2—C3	122.8 (2)	S2—C12—H12B	108.9
N1—C2—H2	118.6	H12A—C12—H12B	107.7
C3—C2—H2	118.6	N5—C13—N4	127.3 (2)
C4—C3—C2	117.5 (2)	N5—C13—S2	119.94 (18)
C4—C3—H3	121.2	N4—C13—S2	112.72 (18)
C2—C3—H3	121.2	N4—C14—C15	124.0 (2)
N2—C4—C3	121.2 (2)	N4—C14—H14	118.0
N2—C4—C5	116.95 (19)	C15—C14—H14	118.0
C3—C4—C5	121.8 (2)	C14—C15—C16	116.9 (2)
C8—C5—C6	117.4 (2)	C14—C15—H15	121.6
C8—C5—C4	122.0 (2)	C16—C15—H15	121.6
C6—C5—C4	120.6 (2)	N5—C16—C15	120.7 (2)
C7—C6—C5	118.4 (2)	N5—C16—C17	116.6 (2)
C7—C6—H6	120.8	C15—C16—C17	122.7 (2)
C5—C6—H6	120.8	C18—C17—C20	116.4 (2)
N3—C7—C6	124.9 (2)	C18—C17—C16	122.6 (2)
N3—C7—H7	117.6	C20—C17—C16	121.0 (2)
C6—C7—H7	117.6	C19—C18—C17	119.9 (2)
C9—C8—C5	119.8 (2)	C19—C18—H18	120.1
C9—C8—H8	120.1	C17—C18—H18	120.1
C5—C8—H8	120.1	N6—C19—C18	124.3 (2)
N3—C9—C8	123.7 (2)	N6—C19—H19	117.8
N3—C9—H9	118.1	C18—C19—H19	117.8
C8—C9—H9	118.1	C21—C20—C17	119.5 (2)
C11—C10—S1	108.09 (17)	C21—C20—H20	120.3
C11—C10—H10A	110.1	C17—C20—H20	120.3
S1—C10—H10A	110.1	N6—C21—C20	124.4 (2)
C11—C10—H10B	110.1	N6—C21—H21	117.8
S1—C10—H10B	110.1	C20—C21—H21	117.8
